# Correction: Factors associated with reluctancy to acquire COVID-19 vaccination: A cross-sectional study in Shiraz, Iran, 2022

**DOI:** 10.1371/journal.pone.0307336

**Published:** 2024-07-12

**Authors:** Najmeh Maharlouei, Parisa Hosseinpour, Amirhossein Erfani, Reza Shahriarirad, Hadi Raeisi Shahraki, Abbas Rezaianzadeh, Kamran Bagheri Lankarani

The fifth author’s name is spelled incorrectly. The correct name is: Hadi Raeisi Shahraki. The correct citation is: Maharlouei N, Hosseinpour P, Erfani A, Shahriarirad R, Raeisi Shahraki H, Rezaianzadeh A, et al. (2022) Factors associated with reluctancy to acquire COVID-19 vaccination: A cross-sectional study in Shiraz, Iran, 2022. PLOS ONE 17(12): e0278967. https://doi.org/10.1371/journal.pone.0278967.

The affiliation for the fifth author is incorrect. The correct affiliation is not indicated. Hadi Raeisi Shahraki is not affiliated with #1 but with: Department of Epidemiology and Biostatistics, Faculty of Health, Shahrekord University of Medical Sciences, Shahrekord, Iran.

## References

[1] Maharlouei N, Hosseinpour P, Erfani A, Shahriarirad R, Raeisi Shahrakie H, Rezaianzadeh A, et al. (2022) Factors associated with reluctancy to acquire COVID-19 vaccination: A cross-sectional study in Shiraz, Iran, 2022. PLOS ONE 17(12): e0278967. 10.1371/journal.pone.0278967 36508442 PMC9744289

