# Supplementary material for: Factors associated with reluctancy to acquire COVID-19 vaccination: A cross-sectional study in Shiraz, Iran, 2022
Source: PLoS One. 2022 Dec 12;17(12):e0278967. doi: 10.1371/journal.pone.0278967 (PMC9744289; doi:10.1371/journal.pone.0278967)
Supplement: Attachment — Submitted filename: Response to Reviewers.docx [file pone.0278967.s001.docx]

**Journal Requirements:**

When submitting your revision, we need you to address these additional requirements.

1. Please ensure that your manuscript meets PLOS ONE's style requirements, including those for file naming. The PLOS ONE style templates can be found at
   <https://journals.plos.org/plosone/s/file?id=wjVg/PLOSOne_formatting_sample_main_body.pdf> and 
   <https://journals.plos.org/plosone/s/file?id=ba62/PLOSOne_formatting_sample_title_authors_affiliations.pdf>

**Authors Response:** We apologize for this inconvenience and have revised the manuscript based on the provided guidelines.

2. We suggest you thoroughly copyedit your manuscript for language usage, spelling, and grammar. If you do not know anyone who can help you do this, you may wish to consider employing a professional scientific editing service.

**Authors Response:** Our manuscript has been revised by a Native English editor and we have attached the certificate for your reference.

3. Upon resubmission, please provide the following:

● The name of the colleague or the details of the professional service that edited your manuscript

 ● A copy of your manuscript showing your changes by either highlighting them or using track changes (uploaded as a *supporting information* file)

 ● A clean copy of the edited manuscript (uploaded as the new *manuscript* file)

**Authors Response:** We have uploaded the mentioned files as requested.

4. Thank you for stating the following in the Acknowledgments Section of your manuscript:
   "This study was the subject of the MPH degree thesis for Parisa Hossseinpour and Amirhossein Erfani. The authors would like to thank the Vice Chancellor for Research of Shiraz University of Medical Sciences for financially supporting the project (Project code: 22574)."

We note that you have provided funding information that is not currently declared in your Funding Statement. However, funding information should not appear in the Acknowledgments section or other areas of your manuscript. We will only publish funding information present in the Funding Statement section of the online submission form. Please remove any funding-related text from the manuscript and let us know how you would like to update your Funding Statement. Currently, your Funding Statement reads as follows: "Vice-chancellor for Research of Shiraz University of Medical Sciences financially supported this Study through Kamran Bagheri Lankarani (Grant No: 24974)."

Please include your amended statements within your cover letter; we will change the online submission form on your behalf.

**Authors Response:** We have moved the funding statement to the cover letter as requested.

5. Your ethics statement should only appear in the Methods section of your manuscript. If your ethics statement is written in any section besides the Methods, please move it to the Methods section and delete it from any other section. Please ensure that your ethics statement is included in your manuscript, as the ethics statement entered into the online submission form will not be published alongside your manuscript.

**Authors Response:** We have moved the ethical consideration section to the method and material section as requested.

6. Please include correct caption for figures.

**Authors Response:** We have adjusted and corrected the figure captions based on the journal’s guidelines.

7. Please review your reference list to ensure that it is complete and correct. If you have cited papers that have been retracted, please include the rationale for doing so in the manuscript text, or remove these references and replace them with relevant current references. Any changes to the reference list should be mentioned in the rebuttal letter that accompanies your revised manuscript. If you need to cite a retracted article, indicate the article’s retracted status in the References list and also include a citation and full reference for the retraction notice.

**Authors Response:** We have revised the manuscript reference list based on the journal’s guidelines.

**Comments to the Author**

*Dear Editor and Reviewers,*

Thanks for reaching out to us regarding the manuscript entitled “Associated Factors of Reluctancy Toward COVID-19 Vaccination; A Cross-Sectional Study in Shiraz, Southern Iran ". We believe that these comments have helped us enhance the quality of the manuscript. We also have done our best to revise and improve the paper according to the comments. Herewith, we provided the authors' responses to each comment right after each statement. Also, all the changes have been addressed in the manuscript through highlighted parts according to journal policies.

- **Reviewer #1**:

1. Include a short note on the origin of COVID-19 (refer and cite: doi: 10.1136/postgradmedj-2020-138234

**Authors’ Response:** Thank you for your comment. A statement regarding the origin of COVID-19 has been added to the manuscript according to the provided reference. (Lines 44 – 51)

2. Compare the COVID-19 states in Iran with other regions (refer and cite: doi: 10.3389/fpubh.2022.844333)

**Author’s Response:** Thank you for your comment and for providing us with this valuable article. A statement has been added to the manuscript addressing the economic effects of COVID-19. (Lines 47 – 51)

3. The role of vaccination status that has declined the vaccine resistance rates(refer and cite: doi: 10.3390/vaccines9101064.)

**Authors’ Response:** Thank you for your comment. This article was referred to in the discussion section regarding the concerns for the post-vaccine scare of adverse health effects. (Discussion; lines 266 – 268)

4. How the Iranian government and health care has imbibed regulations to combat COVID-19 targeting its predictors (refer and cite: doi: 10.1136/postgradmedj-2021-141365)

**Authors’ Response:** Thank you for the valuable comment. Since combating vaccine hesitancy might differ from one society to another, a statement regarding considering cultural beliefs and educational status was added to the manuscript. (Paragraph 2 Discussion section; lines 263 – 265; and Paragraph 4 discussion section; line 281 – 283)

5. The treatment of COVID-19 and its implications on the vaccine hesitancy (refer and cite: doi: 10.3389/fphar.2022.742273.

**Authors’ Response:** Thank you for your comment. Although we did not evaluate the effect of treatment on vaccine hesitancy, we have added this statement in the limitation section and also a statement regarding the practice of available medicines in combating the COVID-19 pandemic has been added to the manuscript (Lines 52 and 53).

6. Include other forms of representations in your figures (eg. Bar charts, histograms), color the images for better viewership.

**Authors’ Response:** Thank you for your comment. Two more figures regarding the reasons of reluctancy toward vaccination and influencing the of participation in vaccination based on socio-economic status has been added to the manuscript (Figure 4 and 5).

- **Reviewer #2:**

1. Grammatical errors need to be corrected.

**Authors’ Response:** Thank you for your valuable comment. The manuscript has been revised in aspect of grammatical errors by a professional English editor.

2. Include illustrations or bar charts.

**Authors’ Response:** Thank you for your comment. Two more figures regarding the reasons of reluctancy toward vaccination and influencing the of participation in vaccination based on socio-economic status has been added to the manuscript (Figure 4 and 5).

3. The introduction is very verbose. Needs to be tapered.

**Authors’ Response:** Thank you for your comment and concern. Based on the first reviewers comments we were obligated to add some additional requested information. Furthermore, we believe that any tapering in the information provided in the introduction section will interrupt the continuity and understanding of the phases and line of thought for the general readers. However, if the honorable reviewer believes that a certain part of the introduction is abundant, we would gladly revise the mentioned section accordingly.
